# Molecular Mechanisms of Cardiac Injury Associated With Myocardial SARS-CoV-2 Infection

**DOI:** 10.3389/fcvm.2021.643958

**Published:** 2022-01-20

**Authors:** Xianfang Liu, Longquan Lou, Lei Zhou

**Affiliations:** ^1^Department of Cardiology, The First Affiliated Hospital of Nanjing Medical University, Nanjing, China; ^2^Department of General Surgery, The Third People's Hospital of Hangzhou, Hangzhou, China

**Keywords:** COVID-19, cardiac injury, SARS-CoV-2, RNA-Seq, bioinformatics analysis

## Abstract

Coronavirus disease 2019 (COVID-19) caused by severe acute respiratory syndrome coronavirus 2 (SARS-CoV-2) has spread around the world. The development of cardiac injury is a common condition in patients with COVID-19, but the pathogenesis remains unclear. The RNA-Seq dataset (GSE150392) comparing expression profiling of mock human induced pluripotent stem cell-derived cardiomyocytes (hiPSC-CMs) and SARS-CoV-2-infected hiPSC-CMs was obtained from Gene Expression Omnibus (GEO). We identified 1,554 differentially expressed genes (DEGs) based on GSE150392. Gene set enrichment analysis (GSEA), Gene ontology (GO) analysis, and Kyoto encyclopedia of genes and genomes (KEGG) pathway analysis showed that immune-inflammatory responses were activated by SARS-CoV-2, while muscle contraction, cellular respiration, and cell cycle of hiPSC-CMs were inhibited. A total of 15 hub genes were identified according to protein–protein interaction (PPI), among which 11 upregulated genes were mainly involved in cytokine activation related to the excessive inflammatory response. Moreover, we identified potential drugs based on these hub genes. In conclusion, SARS-CoV-2 infection of cardiomyocytes caused a strong defensive response, leading to excessive immune inflammation, cell hypoxia, functional contractility reduction, and apoptosis, ultimately resulting in myocardial injury.

## Introduction

Coronavirus disease 2019 (COVID-19) is a viral pandemic caused by the severe acute respiratory syndrome coronavirus 2 (SARS-CoV-2) (1). In December 2019, COVID-19 was first reported in Wuhan of China as a severe unknown form of pneumonia. Subsequently, a global pandemic was declared by the WHO in March 2020. At the time of preparing this manuscript, there were more than 5,00,000 fatalities caused by COVID-19.

Acute myocardial damage is the most common described cardiovascular (CV) complication in patients with COVID-19 (2). In multivariable-adjusted models, cardiac injury has been identified as a significant and independent risk factor [hazard ratios (HR) = 4.26] associated with mortality (3). Cytokine storms (caused by acute systemic inflammation) (1, 4), hypoxemia (5), and pathogen-mediated damage (6, 7) were considered as the potential mechanisms responsible for CV complications in COVID-19. However, the exact mechanism of SARS-CoV-2 infection-related myocardial injury remains unclear.

The study of Arun Sharma et al. reported that SARS-CoV-2 directly infected human induced pluripotent stem cell-derived cardiomyocytes (hiPSC-CMs) *in vitro*, inducing contractility depletion, and apoptosis (8). They obtained the expression profiling of three SARS-CoV-2 infected hiPSC-CMs samples and three mock hiPSC-CMs samples by high throughput sequencing and deposit them on the National Center for Biotechnology Information (NCBI, USA) Gene Expression Omnibus (GEO) database (GSE150392). In this study, we performed a detailed bioinformatics analysis of the GSE150392 RNA-seq data to further examine the specific mechanisms of myocardial damage caused by SARS-CoV-2.

## Materials and Methods

### Data Sources

We obtained the RNA-seq data (GSE150392) from the National Center for Biotechnology Information (NCBI) Gene Expression Omnibus GEO database. The GSE150392 dataset consists of six samples divided into two groups. The Cov group included three hiPSC-CMs (human-induced pluripotent stem cell-derived cardiomyocytes) samples pretreated with SARS-COV-2 for 72 h (Cov-1-3: GSM4548303-5), and the Mock group included three hiPSC-CMs samples with a mock treatment without virus (Mock1-3: GSM4548306-8).

### Identification of Differentially Expressed Genes

We used R (version 4.0.1, https://www.R-project.org/) package “DESeq2” (version 1.28.1) to determine differentially expressed genes (DEGs) (9) (*P*adj <0.01), | (log2FoldChange | > 2) between SARS-CoV-2 infected hiPSC-CMs (Cov group) and Mock hiPSC-CMs (Mock group). The R package “ggplot2” was used to graph the Gene expression values boxplot and minus-vs.-add (MA) plot. *P*adj is the *p*-value using the Benjamini-Hochberg procedure correction, i.e., false discovery rate (FDR).

### Gene Set Enrichment Analysis of All Detected Genes

Gene set enrichment analysis (GSEA, a joint project of UC San Diego and Broad Institute, USA) was performed using the GSEA software (version 4.0.3) implementation in our study for identifying potential hallmarks of SARS-CoV-2 infected hiPSC-CMs (10). The annotated gene sets of “h.all.v7.1.symbols.gmt” were adopted from The Molecular Signatures Database (MSigDB, a joint project of UC San Diego and Broad Institute, USA; http://www.broad.mit.edu/gsea/msigdb/index.jsp). The collapse dataset to gene symbols was “False.” The permutation type was “gene set.” Other parameters were set to default values. Gene sets were considered statistically significant when nominal *p*-value <0.05, FDR was <0.05, and normalized enrichment score (NES) > 1.5.

### Enrichment Analyses of DEGs

Gene ontology (GO) analysis (biological processes, molecular function, and cellular component) and Kyoto encyclopedia of genes and genomes (KEGG) pathway enrichment were analyzed using R package clusterProfiler (version 3.16) (11). Gene ontology analysis (Immune System Process), KEGG pathways functionally grouped networks, and ClinVar human diseases analysis using the CluGO (version 2.5.7) (12) and CluePedia (version 1.5.7) (13) apps of Cytoscape Software (version 3.8) (14). After inputting DEGs in CluGO, select an analysis method in “Ontologies/Pathways” with the following settings: Statistical Test Used was “Enrichment/Depletion (Two-sided hypergeometric test),” Correction Method Used was “Benjamini-Hochberg,” GO Fusion was “false,” and GO Group was “true.” The one with the smallest *P*adj value was retained among the results of the same GO group. *P*adj <0.05 was considered as the cut-off criteria.

### Protein–Protein Interaction Network

The online tool of a search tool for the retrieval of interacting genes (STRING, Swiss Institute of Bioinformatics, Switzerland; https://string-db.org) (15) was applied to establish a Protein–Protein Interactions (PPIs) of DEGs with the score (median confidence) >0.4. Cytoscape and CytoHubba (version 0.1) (16) were used to visualize the PPI network and identify hub genes (Top 15). GeneMANIA online database (https://genemania.org/) was used to analyze the hub genes (17).

### Identifying Drug Candidates

We used the Drug Signatures database DSigDB (University of Colorado at Denver and Health Sciences Center, USA) tool of Enrichr (https://maayanlab.cloud/Enrichr/) to identify drug candidates based on hub genes (18). Drug candidates are ranked by a combined score from highest to lowest.

## Results

### Severe Acute Respiratory Syndrome Coronavirus 2 Infection Caused a Large Number of Gene Expression Changes in hiPSC-CMs

**Figure 1:**
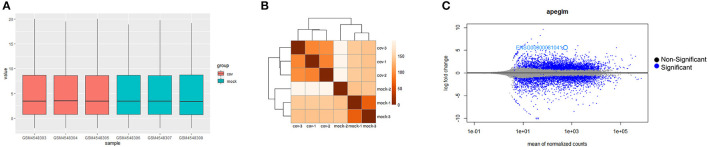
Differential expression of data between two sets of samples. **(A)** Gene expression values boxplot of each sample after normalization using DESeq method; **(B)** Sample to sample distance heatmap of all samples in Cov and Mock group; **(C)** MA plot of genes (The blue points represent genes screened based on |fold change| > 2 and a corrected *P*adj of < 0.01).

The boxplot of expression values of all transcripts indicated similar whole transcriptome expression in each sample (Figure 1A) and sample to sample distance heatmap revealed a relatively clear distinction between samples in the two groups (Figure 1B). Based on the criteria of *P*adj <0.01, and |logFC(fold change)| > 2 (Figure 1C), a total of 1,554 DEGs was identified from GSE150392, including 726 upregulated genes and 828 downregulated genes. Sharma et al. (8) performed a similar DEGs analysis in their previous study, which was not significantly different from the results of this study.

### Gene Set Enrichment Analysis Showed the SARS-CoV-2 Infection Was Mainly Associated With Immune-Inflammatory Response Activity

**Figure 2:**
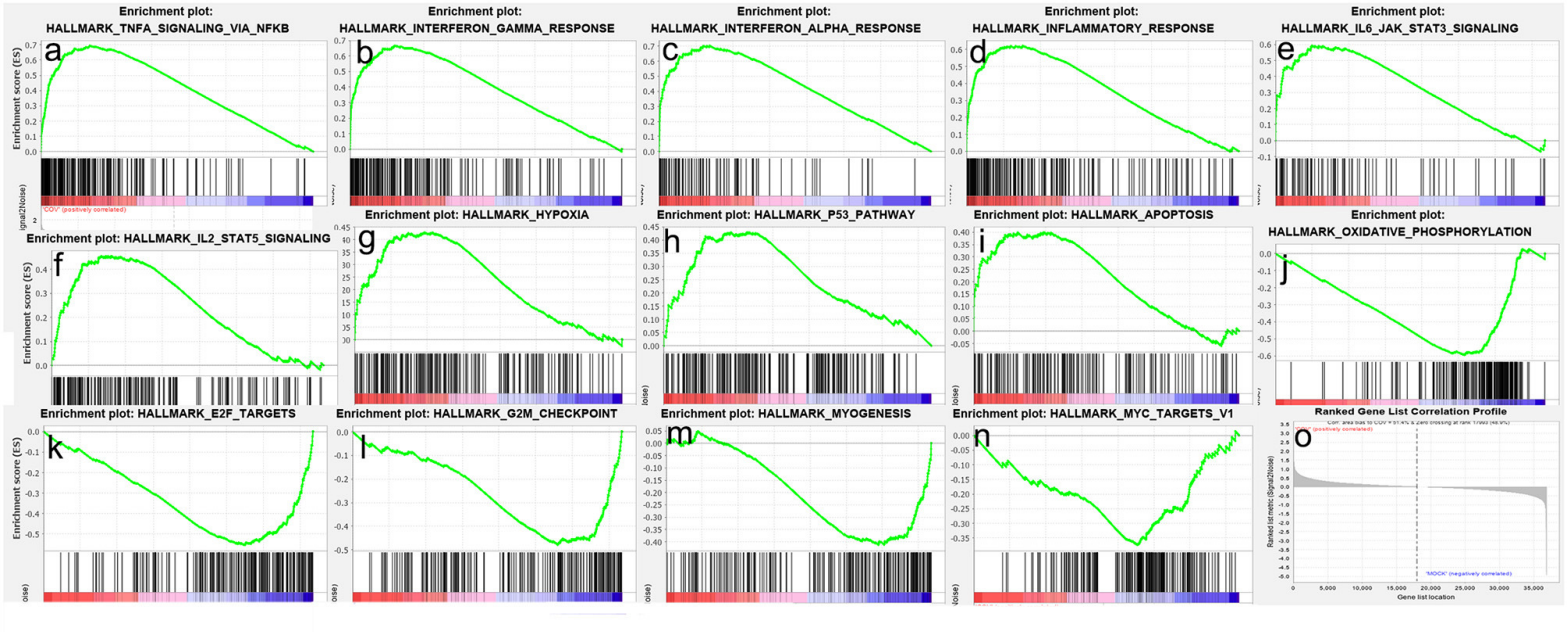
Gene set enrichment analysis (GSEA) of SARS-CoV-2 infection in hiPSC-CMs. **(a–i)** Nine representative gene sets enriched in hiPSC-CMs with SARS-CoV-2 infection; **(j–n)** Five representative gene sets inhibited in hiPSC-CMs with SARS-CoV-2 infection; **(o)** Ranked gene list correlation profile. The red color indicates the “COV” group, the blue color indicates the “MOCK” group, the black line indicates the genes in the GENE SET, and the green line consists of the enrichment fraction of each gene.

To obtain insight into the effect of SARS-CoV-2 on the heart, GSEA was used to map into hallmarks between two groups (Cov vs Mock), 14 significant gene sets were shown (Table 1; Figures 2a–o). Gene set enrichment analysis demonstrated that many inflammation-related gene sets, such as TNFα signaling *via* NF-κB, interferon-γ response, interferon-α response, inflammatory response, IL6-JAK-STAT3 signaling, IL2-STAT5 signaling, hypoxia, P53 pathway, and apoptosis gene sets were enriched by SARS-CoV-2 infection in hiPSC-CMs. Oxidative phosphorylation, E2F targets, G2M checkpoint, myogenesis, and MYC targets v1 gene sets associated with cell cycle, aerobic respiration, and myogenesis were inhibited in hiPSC-CMs with SARS-CoV-2 infection.

**Table 1:** The most significant gene sets of Cov vs. Mock in gene set enrichment analysis (GSEA).

**Gene set follow link to MSigDB**	**NES**	***P*adj**
HALLMARK_TNFA_SIGNALING_VIA_NFKB	2.81	0
HALLMARK_INTERFERON_GAMMA_RESPONSE	2.69	0
HALLMARK_INTERFERON_ALPHA_RESPONSE	2.56	0
HALLMARK_INFLAMMATORY_RESPONSE	2.53	0
HALLMARK_IL6_JAK_STAT3_SIGNALING	2.15	0
HALLMARK_IL2_STAT5_SIGNALING	1.85	4.36E-04
HALLMARK_HYPOXIA	1.73	1.02E-02
HALLMARK_P53_PATHWAY	1.73	9.44E-04
HALLMARK_APOPTOSIS	1.57	6.50E-02
HALLMARK_OXIDATIVE_PHOSPHORYLATION	−2.48	0
HALLMARK_E2F_TARGETS	−2.29	0
HALLMARK_G2M_CHECKPOINT	−2.00	0
HALLMARK_MYOGENESIS	−1.70	3.65E-02
HALLMARK_MYC_TARGETS_V1	−1.57	0.01

*NES, normalized enrichment score*.

### Gene Ontology Term Enrichment Analyses and ClinVar Human Diseases Analysis of DEGs

**Figure 3:**
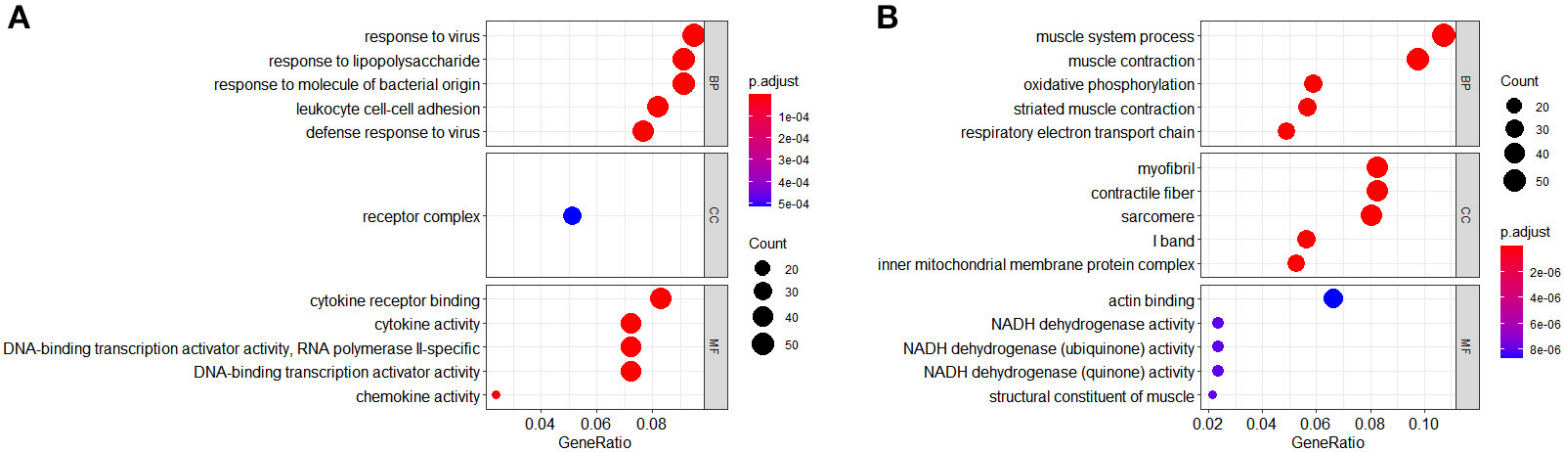
Gene ontology (GO) analysis (BP, CC, and MF) of differentially expressed genes (DEGs). **(A)** GO terms of upregulated DEGs. **(B)** GO terms of downregulated DEGs.

Gene ontology analysis of DEGs was divided into four functional groups, including biological processes (BP), cell composition (CC), molecular function (MF), and Immune System Process (ISP). The top five results of BP, CC, and MF are shown in Figure 3.

In the BP group, the upregulated genes were mainly enriched in response to the virus, response to lipopolysaccharide, response to molecule of bacterial origin, leukocyte cell–cell adhesion, and defense response to the virus. The downregulated genes were mainly concentrated in the muscle system process, muscle contraction, oxidative phosphorylation, striated muscle contraction, and respiratory electron transport chain.

In the CC group, the upregulated genes were mainly enriched in receptor complex. The downregulated genes were mainly concentrated in myofibril, contractile fiber, sarcomere, I band, and the inner mitochondrial membrane protein complex.

In the MF group, the upregulated genes were mainly involved in terms of cytokine receptor binding, cytokine activity, DNA-binding transcription activator activity (RNA polymerase II-specific), DNA-binding transcription activator activity, and chemokine activity. The downregulated genes were mainly enriched in actin binding, NADH dehydrogenase activity, NADH dehydrogenase (ubiquinone) activity, NADH dehydrogenase (quinone) activity, and structural constituent of muscle.

The top five ISP results were shown in Table 2. Type I interferon signaling pathway, regulation of adaptive immune response, neutrophil migration, regulation of type 2 immune response, and CD4-positive, α-β T cell cytokine production were enriched by upregulated genes. Positive regulation of megakaryocyte differentiation, regulation of megakaryocyte differentiation, and thymus development were enriched by downregulated genes.

**Table 2:** Gene ontology (GO) (immune system process) analysis of differentially expressed genes (DEGs).

**Term**	**Description**	***P*adj**
**Upregulated genes**		
GO:0060337	Type I interferon signaling pathway	1.02E-08
GO:0002819	Regulation of adaptive immune response	6.53E-05
GO:1990266	Neutrophil migration	2.32E-04
GO:0002828	Regulation of type 2 immune response	2.67E-04
GO:0035743	CD4-positive, alpha-beta T cell cytokine production	8.47E-04
**Downregulated genes**		
GO:0045654	Positive regulation of megakaryocyte differentiation	1.45E-03
GO:0045652	Regulation of megakaryocyte differentiation	1.88E-03
GO:0048538	Thymus development	4.20E-02

ClinVar human diseases analysis indicates that DEGs caused by SARS-CoV-2 infection are significantly involved in terms of myocardial infarction 1, non-syndromic hearing loss and deafness, cardiomyopathy, limb-girdle muscular dystrophy, mitochondrial complex IV deficiency, myofibrillar myopathy, and primary ciliary dyskinesia (Table 3).

**Table 3:** ClinVar human diseases analysis of DEGs.

**Term**	**Description**	***P*adj**
**Upregulated genes**		
C1832662	Myocardial infarction 1	0.02
CN043648	Non-syndromic hearing loss and deafness	0.03
**Downregulated genes**		
C0878544	Cardiomyopathy	6.57E-15
C0686353	Limb-girdle muscular dystrophy	2.47E-04
C0268237	Mitochondrial complex IV deficiency	5.69E-04
C2678065	Myofibrillar myopathy	1.06E-03
C0008780	Primary ciliary dyskinesia	3.32E-02

### KEGG Enrichment Analysis of DEGs

**Figure 4:**
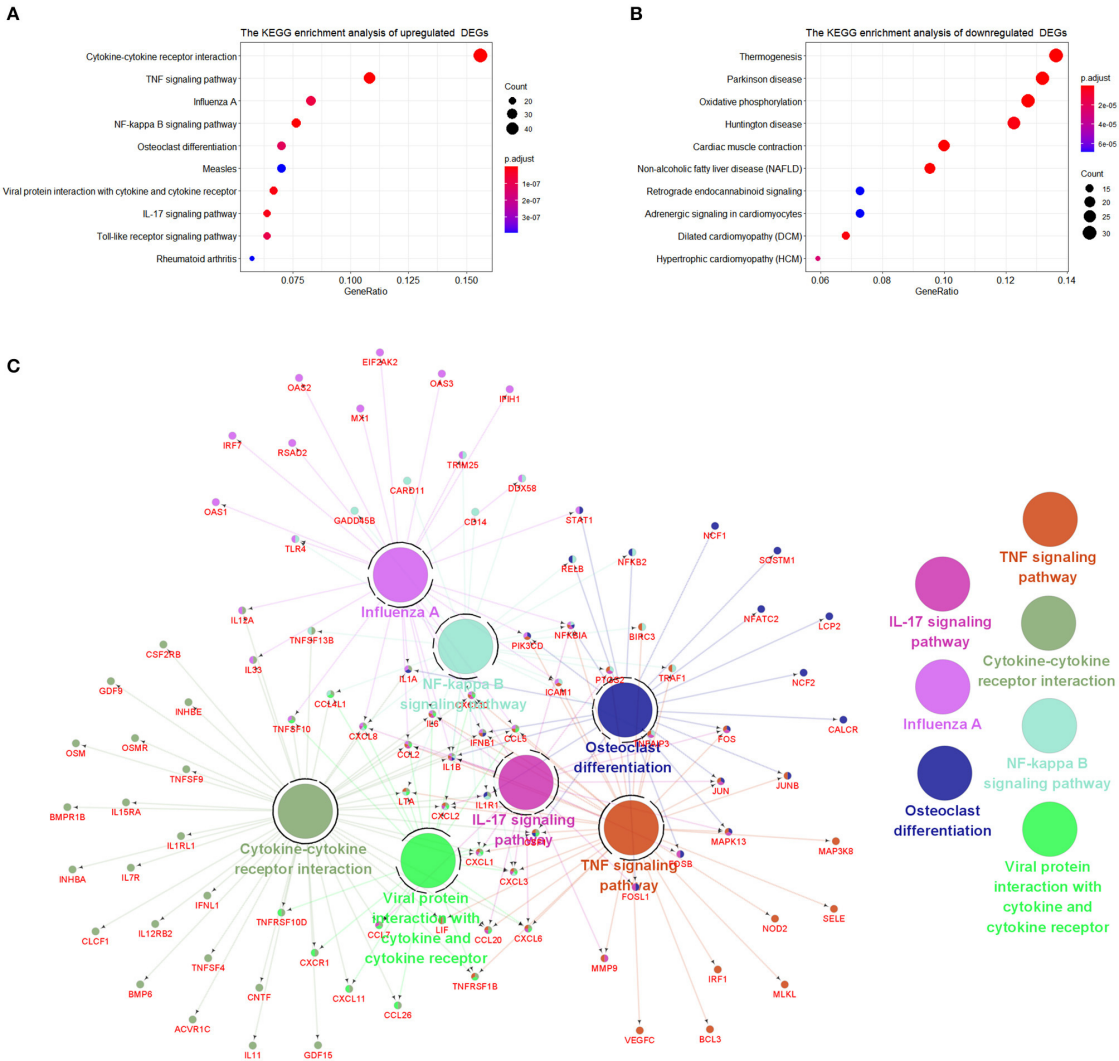
The Kyoto encyclopedia of genes and genomes (KEGG) enrichment analysis of DEGs. **(A)** KEGG pathways of upregulated DEGs. **(B)** KEGG pathways of downregulated DEGs. **(C)** Representative pathways to genes network of upregulated DEGs validated genes (in red) targeted by KEGG pathways.

The signaling pathways of DEGs were shown in Figure 4. The data were imported into Cytoscape to calculate the topological characteristics of the network and determine each node. Cytokine–cytokine receptor interaction, TNF signaling pathway, influenza A, NF-κB signaling pathway, osteoclast differentiation, measles, viral–protein interaction with cytokine and cytokine receptor, IL-17 signaling pathway, Toll-like receptor signaling pathway, and rheumatoid arthritis were significantly enriched by upregulated DEGs (Figure 4A). Thermogenesis, Parkinson's disease, oxidative phosphorylation, Huntington disease, cardiac muscle contraction, non-alcoholic fatty liver disease (NAFLD), retrograde endocannabinoid signaling, adrenergic signaling in cardiomyocytes, dilated cardiomyopathy (DCM), and hypertrophic cardiomyopathy (HCM) were significantly enriched by downregulated DEGs (Figure 4B). The top seven *p*-value KEGG pathways with their target genes were shown (Figure 4C).

### PPI Network Analysis and Hub Genes Recognition

**Figure 5:**
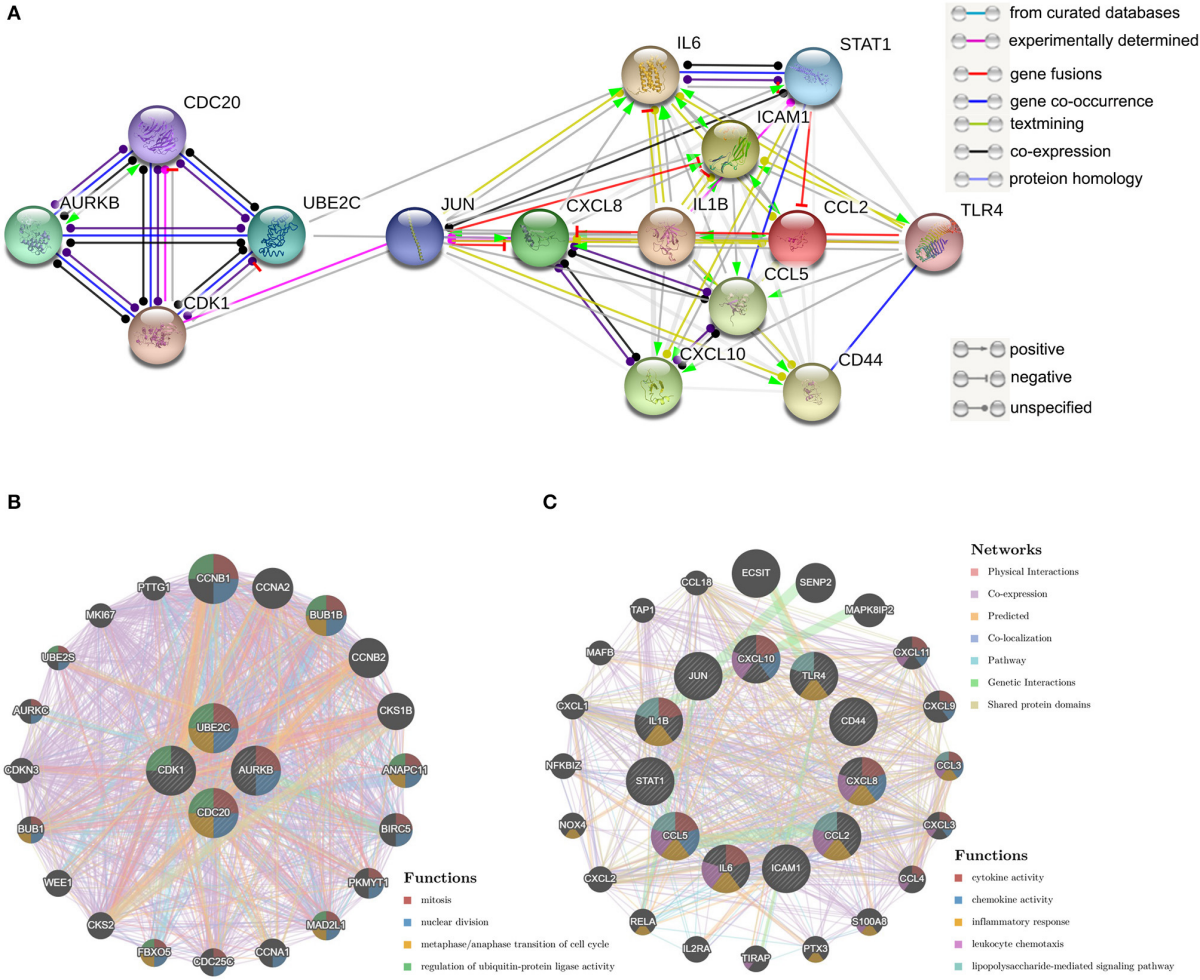
Protein–protein interaction (PPI) network of hub genes. **(A)** The PPI network of the top 15 hub genes created by STRING; **(B)** PP1 networks and function analyses of the four downregulated hub genes; **(C)** PPI networks and function analyses of the 11 upregulated hub genes. Inner circles represent the input genes and outer circles correspond to GeneMANIA proposed hub genes, the size of the circles indicates the correlation with the input genes.

The top 15 genes with the highest interaction degrees were identified, including 11 upregulated genes and four downregulated genes (Table 4). Then those genes were used for constructing the PPI network (Figure 5A). A total of 11 upregulated hub genes showed the complex PPI network with the co-expression of 66.42%, co-localization of 9.39%, genetic interactions of 8.72%, predicted of 8.6%, shared protein domains of 3.99%, pathway of 1.8%, and physical interactions of 1.09% (Figure 5C). Cytokine activity, chemokine activity, inflammatory response, leukocyte chemotaxis, and lipopolysaccharide-mediated signaling pathway were identified as the main function of those genes. Furthermore, four downregulated hub genes showed the complex PPI network with the physical interactions of 67.64%, co-expression of 13.50%, predicted of 6.35%, co-localization of 6.17%, the pathway of 4.35%, genetic interactions of 1.4%, and shared protein domains of 0.59% (Figure 5B). Those genes were related to mitosis, nuclear division, metaphase/anaphase transition of the cell cycle, and regulation of ubiquitin–protein ligase activity.

**Table 4:** Top 15 hub genes with the highest interaction degrees in protein–protein interaction (PPI) network analysis.

**Gene ID**	**Description**
**Upregulated genes**	
IL-6	Interleukin 6
CXCL8	C-X-C motif chemokine ligand 8
TLR4	Toll like receptor 4
STAT1	Signal transducer and activator of transcription 1
IL-1B	Interleukin 1 beta
CXCL10	C-X-C motif chemokine ligand 10
ICAM1	Intercellular adhesion molecule 1
JUN	Jun proto-oncogene, AP-1 transcription factor subunit
CCL5	C-C motif chemokine ligand 5
CCL2	C-C motif chemokine ligand 2
CD44	CD44 molecule
**Downregulated genes**	
CDK1	Cyclin dependent kinase 1
UBE2C	Ubiquitin conjugating enzyme E2 C
CDC20	Cell division cycle 20
AURKB	Aurora kinase B

### Identification of Candidate Drugs

The study further involved 15 hub genes that were used to screen potential drugs for the treatment of SARS-CoV-2 infection-related myocardial injury. According to the combined score, the top 10 candidate drugs were generated (Table 5). Acetovanillone, antimycin A, proscillaridin, chromium (III) chloride, and hyperforin were identified by 11 upregulated hub genes. Irinotecan hydrochloride, troglitazone, 67526-95-8 (thapsigargin), genistein, and testosterone were identified by four downregulated hub genes.

**Table 5:** Drug candidates combined with hub genes.

**Drug name**	***P*adj**	**Combined score**	**Genes**
**Upregulated hub genes**			
Acetovanillone CTD 00002374	6.75E-14	35430.08	CXCL10; JUN; CXCL8; STAT1; CCL5; CCL2; ICAM1
Antimycin A CTD 00005427	3.86E-10	21794.77	JUN; CXCL8; CCL5; CCL2; ICAM1
Proscillaridin CTD 00006639	7.50E-07	16586.61	CXCL8; CCL5; CCL2
Chromium (III) Chloride CTD 00001073	7.50E-07	16586.61	CXCL8; CCL2; ICAM1
Hyperforin CTD 00000051	9.25E-07	14503.67	JUN; CXCL8; ICAM1
**Downregulated hub genes**			
Irinotecan hydrochloride CTD 00002224	2.16E-04	1109900.66	CDC20; UBE2C; CDK1; AURKB
Troglitazone CTD 00002415	2.33E-04	1061016.69	CDC20; UBE2C; CDK1; AURKB
67526-95-8 CTD 00007263	5.84E-04	928089.07	CDC20; UBE2C; CDK1; AURKB
Genistein CTD 00007324	8.24E-04	837567.36	CDC20; UBE2C; CDK1; AURKB
Testosterone CTD 00006844	8.24E-04	832973.85	CDC20; UBE2C; CDK1; AURKB

## Discussion

Severe acute respiratory syndrome coronavirus 2, SARS-CoV, and MERS-CoV (Middle East respiratory syndrome coronavirus) are enveloped positive RNA viruses, belong to the Coronaviridae family β genera (19, 20). Severe acute respiratory syndrome coronavirus 2 and SARS-CoV utilize the same receptor angiotensin-converting enzyme 2 (ACE2) for invading human bodies. Those coronaviruses spread widely among people and lead to potentially fatal diseases (21, 22). Coronavirus disease 2019 caused by SARS-CoV-2 has been spreading in 219 countries/areas/territories, with over 50,000,000 confirmed cases (WHO statistics as of November 9, 2020) (23).

The cardiac injury in COVID-19 was generally defined as the elevation of cardiac-specific biomarkers (e.g., troponin concentration above the 99th percentile upper reference limit) (1, 2). It occurs in roughly 8–13% (24, 25) in confirmed cases and as high as 23–44% in severe patients (26–30). Studies have shown that mortality was markedly higher in patients with cardiac injury than in patients without cardiac injury (51.2–59.6 vs. 4.5–8.9%) (3, 31). Besides, patients with underlying cardiovascular disease (CVD) are more likely to develop acute myocardial injury (29, 31). Concurrent occurrence of underlying CVD and myocardial injury causes significantly higher mortality than patients with underlying CVD but without cardiac injury (69.4 vs. 13.3%) (31), suggesting that myocardial injury played a greater role in the fatal outcome of COVID-19 than the presence of underlying CVD itself.

Angiotensin-converting enzyme 2 is an important target for SARS-CoV and SARS-CoV-2 infection, and the heart is one of the organs which express ACE2 (32). During the SARS epidemic, the SARS-CoV viral RNA was detected on autopsied human heart samples (33). Similarly, Guido Tavazzi et al. reported the first case of myocardial localization of SARS-CoV-2 (34). Furthermore, hiPSC-CMs are susceptible to SARS-CoV-2 infection *in vitro* (8). Those studies all suggested that SARS-CoV-2 may directly infect the heart in the human body through ACE2. However, the pathogenesis of SARS-CoV-2 infection-related acute myocardial injury is still unknown. Previous studies suggested that pathogen-mediated direct myocardial injury, acute systemic inflammatory response (1, 4), and low blood oxygen levels (7) may be the most critical causes of myocardial damage in patients with COVID-19. In this study, we described several specific mechanisms of cardiac injury caused by SARS-CoV-2 direct infection.

We used R software and bioinformatics to deeply analyze the RNA-Seq dataset GSE150293, which compared the gene expression between hiPSC-CMs and SARS-CoV-2-infected hiPSC-CMs samples. The results identified 1554 DEGs, including 726 upregulated genes and 828 downregulated genes. In order to reduce the bias, we first performed a GSEA analysis of all detected genes. Gene set enrichment analysis revealed that SARS-CoV-2 infection activated the inflammatory response, and inhibited oxidative phosphorylation and myogenesis, resulting in hypoxia and apoptosis in cardiomyocytes. Subsequently, GO analysis of DEGs revealed specific mechanisms of SARS-CoV-2 impairing myocardial function. Gene ontology analysis of upregulated DEGs suggested several specific mechanisms of inflammatory response activation, including promotion of receptor complexes expression, enhancement of cytokine and chemokine activity, promotion of cytokine binding to receptors, and boosted leukocyte adhesion. Gene ontology analysis of downregulated DEGs showed muscle system process, muscle contraction, oxidative phosphorylation, striated muscle contraction, and respiratory electron transport chain were significantly inhibited by SARS-CoV-2 infection. The expression of myofibril, contractile fiber, sarcomere, I band, and inner mitochondrial membrane protein complex was also suppressed. In the MF group, SARS-CoV-2 inhibits actin binding, NADH dehydrogenase (such as ubiquinone and quinone) activity, and structural constituent of muscle. Kyoto encyclopedia of genes and genomes pathway analysis showed thermogenesis, oxidative phosphorylation, cardiac muscle contraction, retrograde endocannabinoid signaling, and adrenergic signaling in cardiomyocytes were enriched with downregulated DEGs. Those results reveal specific mechanisms by which SARS-CoV-2 infection causes respiration dysfunction and muscle contraction disorders in hiPSC-CMs.

Previous studies have shown that SARS-CoV-2 infection activates multiple immune responses (35, 36), and to clarify the specific immune response processes involved, we performed ISP enrichment analysis. Similar to previous studies, ISP analysis of upregulated DEGs indicated that adaptive and type-2 immune responses were activated by SARS-CoV-2. In addition, neutrophil migration and CD4-positive α-β T cell cytokine production were promoted. Similarly, GSEA and KEGG pathway analysis showed that immune response correlative signal pathways were activated by SARS-CoV-2, including TNFα, IL6-JAK-STAT3, IL2-STAT5, NF-κB, IL17, and Toll-like receptor signaling pathway. Consistent with the inflammatory cytokines detected in the blood of patients with COVID-19 (1, 37), hiPSC-CMs produce and activate various cytokines, including TNFα, chemokines, interleukins (IL), and interferons after SARS-CoV-2 infection. The massive cytokine release reflects an excessive immune defense, which may cause harmful heart damage. These findings complement our understanding of the human immune response mechanism triggered by SARS-CoV-2 (35) and contribute to the treatment of patients with COVID-19. Similar to our study, Omar Pacha et al. suggested IL17 is immunologically plausible as a target to prevent ARDS in COVID-19 (38).

Furthermore, ISP analysis of downregulated DEGs showed positive regulation of megakaryocyte differentiation, and thymus development were repressed by SARS-CoV-2 infection. These mechanisms may be responsible for the significant reduction of platelet count (39) and peripheral blood T cells (37) in patients with severe COVID-19.

After removing confounding results of genetic disorders, ClinVar human diseases analysis showed SARS-CoV-2 infection may be associated with myocardial infarction (40, 41) and cardiomyopathy (42), which are similar to the clinically reported cardiac complications in patients with COVID-19. Cardiac injury often results in chronic heart failure due to the loss of cardiomyocytes (43). Therefore, we speculate that some patients with COVID-19 combined with myocardial damage may have long-term cardiac insufficiency after recovery.

The top genes with the highest degree of interaction in the PPI network are considered to be hub genes. The identification of hub genes may be critical for the diagnosis and treatment of myocardial injury in patients with COVID-19. In this study, functional analysis of 11 upregulated hub genes showed association with cytokine activity, chemokine activity, inflammatory response, leukocyte chemotaxis, and lipopolysaccharide-mediated signaling pathway. These results were highly similar to the GO analysis of all upregulated DEGs, suggesting that these 11 upregulated hub genes play an irreplaceable role in the SARS-CoV-2-induced myocardial injury process.

It is generally accepted that the regeneration of cardiomyocytes was highest in human infants (44, 45) and declines to relative stagnation in adults (46, 47). Restoring the mitotic activity of cardiomyocytes is considered to be the ultimate solution for the treatment of irreversible heart failure (48). The up-regulation of positive cell cycle regulators is one of the endogenous mechanisms for cardiomyocyte proliferation and regeneration (49). Adenoviral transfection of E2F2 expression in mouse cardiomyocytes leads to the proliferation of adult cardiomyocytes (50). Overexpression of G2/M transition promoter cyclin A2 in ischemic porcine hearts promotes recovery after ischemic injury and induced cytokinesis (51). In this study, GSEA showed that cell cycle-related gene sets such as E2F targets, G2/M checkpoint, myogenesis, and MYC targets v1 were inhibited by SARS-CoV-2 infection. The analysis of the PPI network showed that SARS-CoV-2 inhibits the regenerative potency of hiPSC-CMs by inhibiting the metaphase/anaphase transition of the mitotic cycle, suggesting that the downregulated four hub genes (CDK1/UBE2C/CDC20/AURKB) may be important genes for myogenesis. More research is needed to explain the role of SARS-CoV-2 infection-related mitotic pathways modification in cardiomyocytes, which may contribute to cardiac function recovery in patients with COVID-19.

This study identified 10 drug molecules by hub genes, including acetovanillone, antimycin A, proscillaridin chromium (III) chloride, hyperforin, irinotecan hydrochloride, troglitazone, thapsigargin, genistein, and testosterone. Among these 10 drugs, the cytotoxic drugs chromium (III) chloride and irinotecan hydrochloride should not be considered as candidates. Antimycin A (52), proscillaridin (53, 54), troglitazone (55, 56), thapsigargin (57, 58), genistein (59, 60), and testosterone (61) were considered as potential drugs for COVID-19 in previous studies. Acetovanillone and hyperforin have not been studied for the treatment of COVID-19. The therapeutic effect of these eight candidate drugs on myocardial injury in COVID-19 needs further study.

Overall, our study suggested that SARS-CoV-2 infection can induce a strong immune-inflammatory response, reduce contractility, increase hypoxia, and induce apoptosis in cardiomyocytes. Excessive inflammatory response, respiration dysfunction, and contraction disorders work together to cause cardiac injury. This study supports that direct infection of cardiomyocytes by SARS-CoV-2 is the primary cause of myocardial injury. It should be noted that the occurrence of myocardial injury may be a sign of SARS-CoV-2 entering the circulation, which also explains the acute kidney injury is more common among patients with cardiac injury (3).

The current study has several limitations. The study included only one available data packet of six hiPSC-CMs samples *in vitro*, which could not fully reflect the effect of SARS-CoV-2 on the heart *in vivo*. Only bioinformatics analysis was conducted in this study, further clinical reports or *in vitro* studies are needed to support our conclusions.

## Data Availability Statement

Publicly available datasets were analyzed in this study. This data can be found here: https://www.ncbi.nlm.nih.gov/geo/query/acc.cgi?acc=GSE150392.

## Author Contributions

XL, LL, and LZ designed analyses and drafted the manuscript. XL and LL performed the bioinformatics analysis. LZ reviewed the manuscript and provided funding support. All authors contributed to the final manuscript.

## Funding

This study was supported by the National Natural Science Foundation of China (Grant/Award Numbers: 81970723).

## Conflict of Interest

The authors declare that the research was conducted in the absence of any commercial or financial relationships that could be construed as a potential conflict of interest.

## Publisher's Note

All claims expressed in this article are solely those of the authors and do not necessarily represent those of their affiliated organizations, or those of the publisher, the editors and the reviewers. Any product that may be evaluated in this article, or claim that may be made by its manufacturer, is not guaranteed or endorsed by the publisher.
